# Changes in grey matter development in autism spectrum disorder

**DOI:** 10.1007/s00429-012-0439-9

**Published:** 2012-07-10

**Authors:** Ellen Greimel, Barbara Nehrkorn, Martin Schulte-Rüther, Gereon R. Fink, Thomas Nickl-Jockschat, Beate Herpertz-Dahlmann, Kerstin Konrad, Simon B. Eickhoff

**Affiliations:** 1 Child Neuropsychology Section, Department of Child and Adolescent Psychiatry, Psychosomatics and Psychotherapy, University Hospital of the RWTH Aachen, Aachen, Germany; 2 Department of Child and Adolescent Psychiatry, Psychosomatics and Psychotherapy, University Hospital of the RWTH Aachen, Aachen, Germany; 3 Cognitive Neurology Section, Institute of Neuroscience and Medicine (INM-3), Research Center Juelich, Juelich, Germany; 4 Department of Child and Adolescent Psychiatry, Psychosomatics and Psychotherapy, University Hospital Munich, Munich, Germany; 5 Department of Neurology, University Hospital Cologne, Cologne, Germany; 6 Department of Psychiatry and Psychotherapy, RWTH Aachen University, Aachen, Germany; 7 JARA-Translational Brain Medicine, Juelich/Aachen, Germany; 8 Institute of Neuroscience and Medicine (INM-1), Research Center Juelich, Juelich, Germany; 9 Institute for Clinical Neuroscience and Medical Psychology, Heinrich-Heine University Düsseldorf, Düsseldorf, Germany

**Keywords:** Brain development, MRI, Voxel-based morphometry, Grey matter, Autism spectrum disorder

## Abstract

Results on grey matter (GM) structural alterations in autism spectrum disorder (ASD) are inconclusive. Moreover, little is known about age effects on brain-structure abnormalities in ASD beyond childhood. Here, we aimed to examine regional GM volumes in a large sample of children, adolescents, and adults with ASD. Magnetic resonance imaging scans were obtained in 47 male ASD subjects and 51 matched healthy controls aged 8–50 years. We used whole-brain voxel-based morphometry to first assess group differences in regional GM volume across age. Moreover, taking a cross-sectional approach, group differences in age effects on regional GM volume were investigated. Compared to controls, ASD subjects showed reduced GM volumes in the anterior cingulate cortex, posterior superior temporal sulcus, and middle temporal gyrus. Investigation of group differences in age effects on regional GM volume revealed complex, region-specific alterations in ASD. While GM volumes in the amygdala, temporoparietal junction, septal nucleus and middle cingulate cortex increased in a negative quadratic fashion in both groups, data indicated that GM volume curves in ASD subjects were shifted to the left along the age axis. Moreover, while GM volume in the right precentral gyrus decreased linearly with age in ASD individuals, GM volume development in controls followed a U-shaped pattern. Based on a large sample, our voxel-based morphometry results on group differences in regional GM volumes help to resolve inconclusive findings from previous studies in ASD. Results on age-related changes of regional GM volumes suggest that ASD is characterized by complex alterations in lifetime trajectories of several brain regions that underpin social-cognitive and motor functions.

**Electronic supplementary material:**

The online version of this article (doi:10.1007/s00429-012-0439-9) contains supplementary material, which is available to authorized users.

## Introduction

Autism spectrum disorder (ASD) is a heterogeneous neurodevelopmental disorder characterised by impairments in social interaction and communication, as well as restrictive interests and behaviour (American Psychiatric Association 1994). Moreover, ASD individuals often exhibit motor deficits and impaired action control (Hughes 1996; Jansiewicz et al. 2006). A growing number of magnetic resonance imaging (MRI) studies implicate abnormal grey-matter (GM) morphology in the aetiology of ASD (Amaral et al. 2008), although results are inconclusive. In particular, such structural alterations have been reported for fronto-temporo-limbic areas, including GM reductions in the superior temporal sulcus (STS) and anterior cingulate cortex (ACC) (Hadjikhani et al. 2006; Haznedar et al. 2000; McAlonan et al. 2005). Many studies have been focused on the amygdala, since abnormalities in this region are proposed to play a central role in the social deficits characteristic for ASD (Schultz 2005). However, conflicting evidence exists as to whether amygdala volume in ASD individuals is decreased, increased or comparable relative to healthy subjects (Haznedar et al. 2000; Pierce et al. 2001; Schumann et al. 2004).

These inconsistent findings on structural aberrations in ASD might be due to several differences between the studies, including the exact specification of the disorder, inclusion criteria, or data acquisition and analysis techniques [e.g., manual tracing vs. voxel-based morphometry (VBM)]. Furthermore, sample sizes are typically relatively small, resulting in reduced statistical power. Finally, inconclusive data might also be related to differences between the age groups investigated. The latter might be particularly important when assessing ASD, as robust evidence for atypical brain maturation in young autistic subjects exists. Based on MRI studies and measures of head circumference as an index of brain size, it has been suggested that ASD individuals show normal (e.g., Courchesne et al. 2001; Hazlett et al. 2005; Dawson et al. 2007) or reduced (Courchesne et al. 2003) brain size at birth, followed by a period of accelerated brain growth during infancy (“brain overgrowth”; for a meta-analysis see Redcay and Courchesne 2005). It remains controversial whether the increase in total brain size compared to typically developing individuals persists into later childhood and adolescence (Amaral et al. 2008; Piven et al. 1996; Schumann et al. 2004).

While a considerable number of studies have reported changes in brain development in ASD during infancy and childhood, the wealth of studies focused on total or lobular brain volume is contrasted by the dearth of knowledge about developmental abnormalities in local brain structures (but see, e.g., Mosconi et al. 2009; Schumann et al. 2004, for reports on amygdala development). Moreover, to date only few studies examined age effects on structural brain development in ASD across a large age span. For example, using a region of interest (ROI)-based manual tracing approach, two cross-sectional MRI studies demonstrated changes in caudate nucleus development in ASD subjects compared to controls beyond adolescence. The first study included participants aged 18–49 years (McAlonan et al. 2002), the second study participants aged 6–24 years (Langen et al. 2009). In both studies, caudate nucleus volume was shown to decrease in controls but not in ASD subjects. Changes in brain growth curves may, at least in part, explain the inconsistent findings on structural changes in ASD, since differences between patients and controls may point in either direction depending on the age group investigated.

The aim of the present MRI study was twofold. First, we aimed to investigate alterations in GM structure in a large sample of ASD subjects (compared to healthy controls) based on user-independent whole-brain VBM. Second, by adopting a cross-sectional VBM approach, we aimed to compare age-related changes in local GM volume in ASD subjects to those of controls across a large age span. We included subjects aged 8–50 years, thereby extending the age range that has been typically investigated in studies on structural brain development in ASD (but see Raznahan et al. 2010). Based on previous findings, we hypothesized that ASD subjects would show GM reductions in the ACC and the STS (McAlonan et al. 2005; Haznedar et al. 2000; Hadjikhani et al. 2006). No directional hypothesis could be formulated as to whether amygdala volume in the ASD group would be increased or decreased compared to controls as the literature has provided conflicting evidence on that issue (Schumann et al. 2004; Pierce et al. 2001; Haznedar et al. 2000). Moreover, we hypothesized that in ASD individuals, GM development of fronto-temporo-limbic brain structures previously implicated in the pathophysiology of ASD, would differ from controls. Building on the few previous results on age-related changes of regional GM volumes as obtained from volumetric studies, we specifically expected that GM development of the amygdala (Mosconi et al. 2009; Schumann et al. 2004) and the caudate nucleus (McAlonan et al. 2002; Langen et al. 2009) would differ from controls.

## Method

### Participants

Forty-seven subjects diagnosed with Asperger syndrome (*n* = 26), high-functioning autism (*n* = 20) or atypical autism (*n* = 1; ICD-10 code F84.1), and 51 healthy controls were studied. Only male subjects with an IQ ≥70 (WISC-III: Wechsler 1991 or WAIS-III: Wechsler 1997) were included. The ASD group included 26 children and adolescents, and 21 adults (age range: 10–50 years). The control group included 29 children and adolescents, and 22 adults (age range: 8–47 years). Age distribution was comparable in both groups (*p* > 0.05). Moreover, groups did not differ significantly in mean age, handedness, full-scale or verbal IQ (Table 1).

**Table 1:** Demographic data of the study sample

	Controls (*n* = 51)	ASD group (*n* = 47)	*p*
Age (mean, SD)	18.3 (7.5)	21.4 (10.1)	NS
Full-scale IQ (mean, SD)	112.5 (12.4)	107.5 (16.6)	NS
Verbal IQ (mean, SD)	111.3 (12.7)	104.2 (22.6)	NS
Performance IQ (mean, SD)	109.6 (12.0)	99.9 (16.4)	<.05
Handedness: no. left/no. right	4/47	5/42	NS

*ASD* autism spectrum disorder, *NS* not significant

Performance IQ was significantly lower in the ASD group, which is typical for a sample including subjects with Asperger syndrome (Klin 2000). To (1) control for and (2) analyze potential effects of IQ on VBM results, full-scale, verbal and performance IQ were entered as covariates in all subsequent analyses (see “Data analysis”).

ASD subjects were diagnosed by experienced clinicians according to ICD-10 (World Health Organization 1993) and DSM-IV (American Psychiatric Association 1994). Diagnoses were confirmed by the Autism Diagnostic Observation Schedule-Generic (ADOS-G) (Lord et al. 2000). Moreover, in ASD subjects aged <18 years, an autism-specific parent interview (Autism Diagnostic Interview-Revised) (LeCouteur et al. 1989) was conducted. ADOS-G and ADI-R were conducted by certified examiners. In addition, parents of ASD participants aged <18 completed the Social Communication Questionnaire (Rutter et al. 2003). ASD participants ≥18 years of age completed the Autism Spectrum Questionnaire (Baron-Cohen et al. 2001). 

In the subject diagnosed with atypical autism, the triad of autistic symptoms (deficits in social interaction, communication, and restrictive interests and behaviour) was present but had been observed slightly after the age of 3 years according to the recollections of the parents. The subject diagnosed with atypical autism did not comprise an outlier in the results. Excluding the subject with atypical autism from the analyses did not result in any change of the observed pattern of results. Moreover, an exploratory analysis did not reveal any significant differences (even at a very liberal level) between subjects with high-functioning autism and subjects with Asperger syndrome in regional GM volumes or age-related changes of regional GM volume. Consequently, data of the three ASD groups were pooled for all further analyses (see Appendix 2 in ESM for details).

In ASD subjects aged <18 years, comorbid diagnoses were screened using the Child Behavior Checklist (CBCL, Achenbach 1993) and the FBB-HKS (German parental report on ADHD symptoms, Döpfner and Lehmkuhl 1998). In ASD subjects aged ≥18 years, the Brief Symptom Inventory (Derogatis 1993) and the ADHD Behavior Checklist for Adults (Murphy and Barkley 1995) were used. Moreover, in all ASD individuals comorbidity was assessed based on an extensive psychiatric, psychological and neurological examination.

With regard to psychiatric comorbidities, six subjects showed symptoms of ADHD, one subject had been diagnosed with chronic tic disorder, and one subject with depressive disorder.

Control subjects were extensively screened to exclude psychiatric disorders using a semi-structured interview (K-SADS-PL) (Kaufman et al. 1997) and the CBCL for children and adolescents, and the Brief Symptom Inventory for adults. Moreover, autistic symptoms in controls were screened based on the Social Communication Questionnaire (in children and adolescents) and the Autism Spectrum Questionnaire (in adults). Control subjects who scored above the relevant thresholds were excluded from the study.

Nine ASD participants were medicated at time of testing (atypical neuroleptics: *n* = 5; typical neuroleptics: *n* = 1; atomoxetine: *n* = 2; selective serotonin–noradrenalin reuptake inhibitor: *n* = 1). A supplementary analysis did not reveal significant differences between unmedicated ASD subjects and medicated ASD subjects in regional GM volumes or age-related changes of regional GM volume. Therefore, data of unmedicated and medicated ASD subjects were pooled for all further analyses (see Appendix 2 in ESM for details).

None of the control subjects received any medication. No participant in either group suffered from any relevant somatic or neurological disorders. The study was approved by the institutional review board of the University Hospital of the RWTH Aachen and has been performed in accordance with the Declaration of Helsinki. All participants were informed about the experimental procedures and aims of the study, and provided written informed consent (subjects aged ≥18 years) or assent (subjects aged <18 years). For children or adolescents, additional written informed consent was obtained by at least one parent/legal custodian, after the parent(s)/legal custodian(s) had been informed about the study.

#### MRI acquisition

The majority of subjects (*n* = 88) were scanned on a 1.5 Tesla Avanto system (Siemens, Erlangen, Germany) using a standard head coil. Due to changes in the equipment, ten subjects were scanned on a 3 Tesla Trio system (Siemens, Erlangen, Germany), again using a standard head coil. The proportion of ASD versus control subjects who were scanned on the two systems did not differ significantly. Importantly, an exploratory analysis based on an ANCOVA with group (ASD vs. controls) as condition and scanner as covariate (1.5 vs. 3 Tesla) revealed neither a main effect of scanner nor an interaction effect of scanner × group on regional GM volume at a threshold of *p* < 0.05, corrected for multiple comparisons. Similarly, when conducting the same analysis with scanner and group as conditions, no significant main effects of scanner or interaction effects of scanner × group on regional GM volumes were revealed, even when lowering the threshold to *p* = 0.001 uncorrected. Moreover, the overall pattern of results remained stable when only the (*n* = 88) subjects who were scanned on the 1.5 Tesla system were considered (for details, see “Results” and Appendix 2 in ESM). Consequently, data from both scanners was pooled for all subsequent analyses.

High-resolution T1-weighed anatomical images were collected using a rapid acquisition gradient-echo pulse sequence (TE = 3.93 ms, TR = 2,200 ms, FOV = 256 mm, matrix size = 256 × 256, voxel size = 1 × 1 × 1 mm^3^).

Throughout the study period, quality assurance was maintained by performing a phantom scan on each measurement day.

### Data analysis

Imaging data were analyzed with SPM5 (Wellcome Department of Imaging Neuroscience, London, UK: http://www.fil.ion.ucl.ac.uk) running on MATLAB 7.2 (The Mathworks, Inc., Natrick, MA, USA). VBM analyses were applied as implemented in SPM5 using the unified segmentation approach. This approach combines tissue classification, spatial normalization and bias correction into an integrated unified generative model (Ashburner and Friston 2005). Structural images were segmented into GM, white matter (WM) and cerebrospinal fluid using tissue prior probability templates provided with SPM5. Segmented and modulated (to preserve total volume) normalized GM images were smoothed with a Gaussian kernel of 8 mm full-width half-maximum to accommodate residual spatial variability after normalization.

Before addressing the main research questions, total brain volume (TBV), and global volumes of GM and WM were calculated and compared between groups using univariate ANCOVAs. Age, age in years squared and IQ (full-scale, verbal and performance IQ) were used as covariates to control for possible confounds.

For analysis of regional GM differences between groups, modulated GM images were entered in a second-level Bayesian mixed effects model to allow inference to the general population. To control for potential confounding variables, TBV, age (linear and quadratic age regressor: age in years and age in years squared, respectively), full-scale, verbal and performance IQ were entered as covariates into the same model. For model estimation, we used the probabilistic empirical Bayes algorithm as implemented in SPM5. The ensuing voxel-wise statistical maps were thresholded at a posterior probability at 0.99, i.e., only those voxels survive thresholding where the confidence for true group differences is >99 % (for details, see Appendix 1 in ESM).

The Bayesian approach was chosen over the classical frequentionist approach, as it circumvents the problem with non-stationary noise (potentially invalidating the assumptions of Gaussian fields modelling) in multiple-comparison correction of VBM data. Bayesian modelling allows the estimation of conditional probabilities for the existence of group differences given the data. Thus, one advantage of Bayesian inference is that it cannot only be used to declare group differences with a quantifiable confidence but also to quantify the likelihood for the absence of differences between groups (Friston et al. 2008; Friston and Penny 2003a). In classical inference, *p* values only give the probability of observing group differences by chance in an infinitive number of experiments, without permitting statements on the probability for genuine regional GM group differences. Another important rationale for using the Bayesian approach is its high face validity, as the inference is made about an effect being greater than a specified threshold (γ threshold, see Appendix 1 in ESM) which relates to the “background noise level”. In contrast, in classical inference, the inference is made about an effect being significantly greater than zero. Given sufficient observations, negligible effects become significant (Friston and Penny 2003a).

To explore differences in age effects on regional GM volume between controls and ASD individuals, modulated GM images were entered into a second-level random effects group analysis using an ANCOVA. Two regressors specifying linear and quadratic effects of age as well as regressors reflecting TBV, full-scale, verbal and performance IQ were included as covariates into the model. All regressors entered the model as mean-corrected by group interactions to orthogonalize them to the group main effects. This allowed to assess the main effects (over groups) and the differential effects (depending on group) of these covariates on GM volumes. F-contrasts were performed to explore differential regression with age. Hereby, we assessed whether parameters for the regional GM volume regression curves on age differed significantly between both groups, i.e., whether there were linear, quadratic or compound effects of age on regional GM volume.

The ‘unit’ of VBM measurements, in turn, represents the probability for GM at each particular voxel after “modulation” to keep the local volume representation constant in spite of the expansion/contraction during spatial normalization. Put differently, the unit can be interpreted as local native GM volume in the respective standard-space voxel. The data entering the regression analyses additionally had variance explained by the group-specific mean GM volume at this peak voxel as well as any variance accounted for by modelled confounds removed.

As the mean GM volume for each group, and the effects of potential confounds (TBV, full-scale, verbal and performance IQ) were partialled out in every single voxel, age-related changes of regional GM volumes were analysed independent of potential between-group differences in regional GM volumes. That is, the analysis of age-related effects was orthogonal to the analysis of differences between the groups in mean GM volumes. Put differently, the analysis of age-related patterns explained additional variance in the data that could not be accounted for by the mean GM density in each group.

To assess the impact of autistic pathology on regional GM volumes (see, e.g., Brieber et al. 2007), we additionally computed a linear regression analysis including all ASD subjects with the ADOS-G total score as the independent variable. TBV, the linear and quadratic age regressors, full-scale, verbal and performance IQ were included as covariates into the regression model.

Results of the regression analyses that met the statistical threshold of *p* < 0.05 (voxel-wise FDR corrected) (Genovese et al. 2002) are reported (extent threshold = 100 voxels).

Results from all analyses were anatomically localised using the SPM Anatomy toolbox (www.fz-juelich.de/ime/spm_anatomy_toolbox) (Eickhoff et al. 2005, 2006, 2007).

## Results

### Group differences in global brain volumes

Univariate ANCOVAs with age, age in years squared and IQ (full-scale, verbal and performance IQ) as covariates did not reveal any significant group differences between controls and ASD individuals with regard to TBV, or global volume of GM and WM (Table 2).

**Table 2:** Global brain volumes

	Controls (*n* = 51)	ASD group (*n* = 47)	*p* ^*a*^
Total brain volume (ml) (mean, SD)	1,402.9 (128.6)	1344.3 (123.9)	NS
Global GM volume (ml) (mean, SD)	870.6 (93.6)	820.3 (98.8)	NS
Global WM volume (ml) (mean, SD)	532.3 (69.8)	524.0 (69.2)	NS

^a^Group differences were tested using univariate ANCOVAs with age, age in years squared and IQ (full-scale, verbal and performance IQ) as covariates

*ASD* autism spectrum disorder, *GM* grey matter, *WM* white matter, *NS* not significant

### Group differences in regional grey matter volumes

**Fig. 1:**
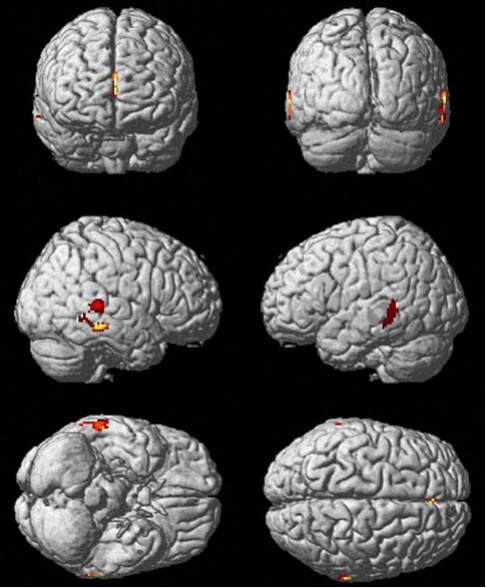
Group differences in regional grey matter volumes. Grey matter (GM) reductions in autism spectrum disorder (ASD) subjects relative to healthy controls in the anterior cingulate cortex, bilateral posterior superior temporal sulcus and right middle temporal gyrus. Areas of significant decrease in GM volume (99 % confidence for group differences greater than the background noise) are shown superimposed on a MNI single-subject template. *Lighter colours* represent higher probabilities for group differences

In ASD subjects, GM volume in the ACC (local maximum at 0, 42, 12; all coordinates in MNI space) was reduced compared to controls. In addition, ASD subjects showed a decrease in GM volume relative to controls in the bilateral posterior STS (local maxima in the right and left middle temporal gyrus; 68, −34, 4/− 66, −44, 6) and right middle temporal gyrus (62, −30, −12) (Fig. 1). Estimates of effect sizes (Cohen’s *d*) for the reported group differences are provided in Appendix 2 in ESM. No GM increases in the ASD group relative to control subjects were observed.

### Group differences in age-related changes of regional grey matter volumes

**Fig. 2:**
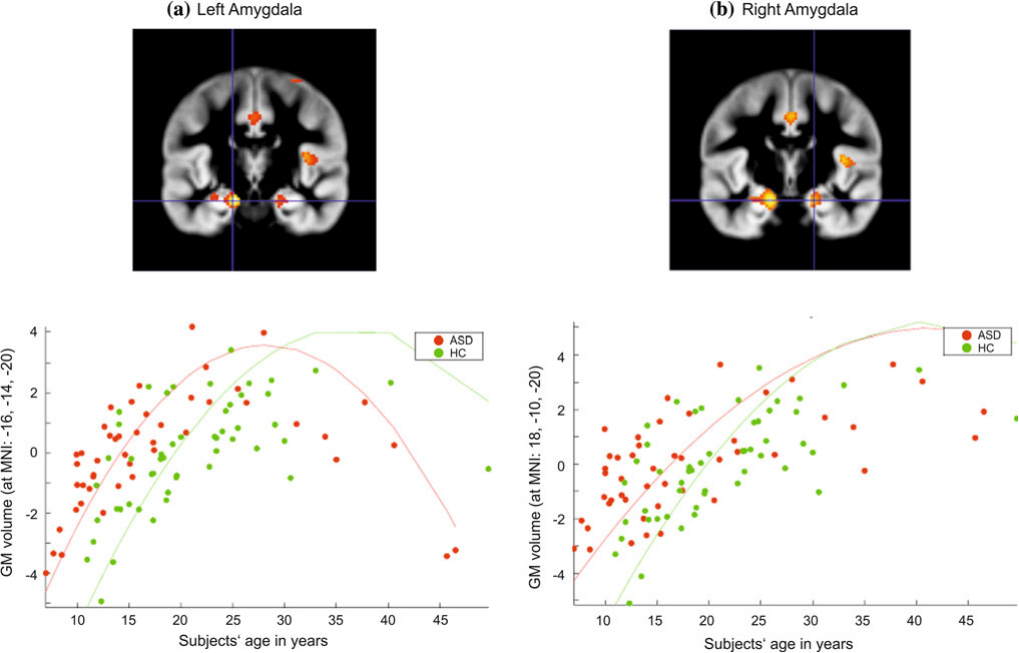
Group differences in age-related changes of regional grey matter volumes. Group differences in age-related regional grey matter volume curves in the **a** left amygdala, **b** right amygdala in individuals with autism spectrum disorder (ASD) compared to healthy controls (HC). Coordinates are given in MNI space. The *y*-*axis* represents regional GM volumes after removing variance explained by the group-specific mean GM volume at this voxel as well as any variance accounted for by modelled confounds. The fitted response plotted for each subject consists of the effects explained by linear or quadratic age-related effects and residual variance. The *curves* represent the results of the second-order regression model (fitted independently to both groups)

**Fig. 3:**
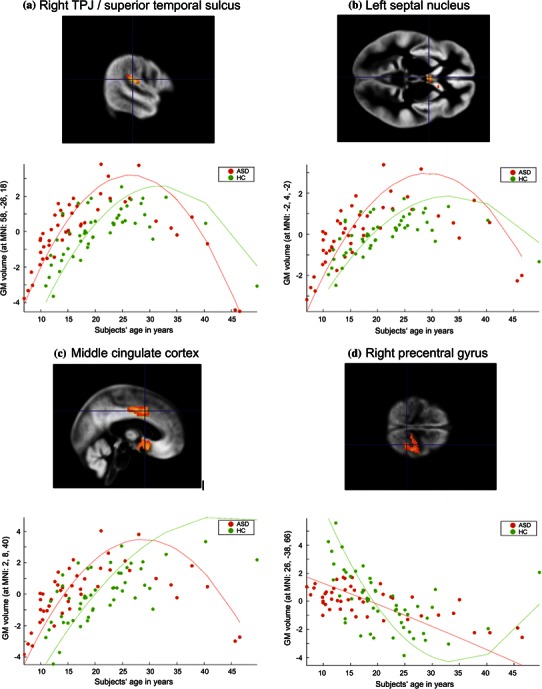
Group differences in age-related changes of regional grey matter volumes. Group differences in age-related regional grey matter volume curves in the **a** right temporoparietal junction (superior temporal gyrus), **b** left septal nucleus, **c** middle cingulate cortex, and **d** left precentral gyrus in individuals with autism spectrum disorder (ASD) compared to healthy controls (HC). Coordinates are given in MNI space. The *y*-*axis* represents regional GM volumes after removing variance explained by the group-specific mean GM volume at this voxel as well as any variance accounted for by modelled confounds. The fitted response plotted for each subject consists of the effects explained by linear or quadratic age-related effects and residual variance. The *curves* represent the results of the second-order regression model (fitted independently to both groups)

Age-related changes in GM volumes of the left amygdala (−16, −14, −20; Fig. 2a), right temporoparietal junction (TJP; local maximum in the superior temporal gyrus; 58, –26, 18; Fig. 3a), and left septal nucleus (−2, 4, −2; Fig. 3b) differed significantly between groups. While GM volumes in these three regions exhibited an inverted U-shaped trajectory in both groups, the curves in ASD subjects were shifted to the left along the age axis compared to the controls.

Divergent age-related changes were also revealed for the right amygdala (18, −10, −20; Fig. 2b). GM volume increased in a negative quadratic fashion until middle adulthood in both groups. Again, the curve in ASD individuals was shifted to the left. In contrast to the pattern described for the left amygdala, however, in the right amygdala, GM volume curves converged with increasing age.

Moreover, age effects on GM volume in the middle cingulate cortex (2, 8, 40; Fig. 3c) significantly differed between groups. During childhood and adolescence, ASD individuals exhibited a curve parallel to controls with a leftward shift along the age axis, diverging further from typical development during early and middle adulthood.

Finally, significant group differences in regional GM volume development were found for the right precentral gyrus (26, −38, 66; Fig. 3d). While GM volume decreased linearly with age in ASD individuals, GM volume development in controls followed a U-shaped pattern.

 Equations for the second-order polynomial fit of the age-related changes in local GM volume for all regions are provided in Appendix 2 in ESM.

### Supplementary analyses of (age-related) group differences in regional grey matter volumes

In an additional analysis, we tested for a left- or rightward shift of the age-related GM trajectories in the ASD group relative to controls. This analysis was performed for all regions showing significant differences between the two groups in the parameters of the second-order polynomial fit, i.e., the left and right amygdala, right TJP, left septal nucleus, middle cingulate cortex and right precentral gyrus. To test for a left/rightward shift of the growth trajectories, we identified, for each ASD subject, the control subjects, most similar in regional GM volume, i.e., most similar in “brain development”. We then compared the chronological age of the respective ASD subject to these controls, taking the minimum distance. That is, for each ASD subject, we computed how different (in age) the most similar (in regional GM volume) control subjects were. In line with the visually apparent leftward shift of the GM trajectories, the mean of these difference values across all subjects was positive for the left and right amygdala, right TJP, left septal nucleus, and middle cingulate cortex. That is, the control subjects most similar in regional GM volume were older than the respective patients, indicating a leftward shift of the GM trajectory in ASD. In a next step, we formally tested whether these differences indicating a leftward shift were indeed statistically significant, i.e., statistically different from zero, using one-sample *t* tests. The results showed significant shifts for all regions but the precentral gyrus (all *p*s < 0.05), providing statistical evidence for a leftward shift of GM volume curves in ASD relative to controls.

In a supplementary analysis, we excluded the oldest subjects (over the age of 40), as particularly few participants (*n* = 5) exceeded this age. The analysis revealed that the results of the age regression remain significant for all six regions described above.

Moreover, a re-analysis of the data, excluding the *n* = 10 subjects who were scanned on the 3 Tesla scanner, revealed that the pattern of results for group differences in regional GM volume across age, and group differences in age effects remained virtually the same at the pre-specified thresholds (for details, see Appendix 2 in ESM).

### Effects of IQ and autistic psychopathology on grey-matter volumes

Across all subjects, no significant main effects of full-scale, verbal or performance IQ on GM volumes were observed. Moreover, no differential effects, i.e., differences in correlation between ASD and control subjects, of IQ (full-scale, verbal or performance IQ, respectively) on GM volumes were found.

The final regression analysis revealed no significant correlations between regional GM volumes and autistic psychopathology (ADOS-G) in the ASD group.

## Discussion

We found evidence for reduced GM volumes in the ACC, posterior STS, and middle temporal gyrus in the ASD compared to the control group. Moreover, in the ASD group, age-related changes of GM volume in the amygdala, TPJ, septal nucleus, middle cingulate cortex, and precentral gyrus differed from controls. Group differences in GM volume and its volume changes with age could not be attributed to potential differences between the ASD and the control group in TBV or IQ, as groups did not differ in these aspects (apart from performance IQ) and we additionally controlled for these variables in our analyses.

### Reduced grey matter volumes in autism spectrum disorder

Our findings on GM abnormalities in ASD individuals provide new insight into the structural alterations in ASD, as sample sizes of previous structural neuroimaging studies were often small and in ROI-based studies, the interpretation thereof was further complicated by problems inherent to rater-dependent methods. Our study included a large number of subjects and used an automated rater-independent VBM approach to solidify and extend previous results.

Reductions of GM volume in the STS and middle temporal gyrus in children and adults with ASD have been reported previously (Hadjikhani et al. 2006; Hyde et al. 2010; McAlonan et al. 2005; Wallace et al. 2010). Functional imaging studies suggest that both regions are implicated in social cognition. The STS forms part of the “social brain” (Brothers 1990) and is sensitive to biological motion cues (Pelphrey and Carter 2008). The middle temporal gyrus has been associated with Theory of Mind and empathy (Mosconi et al. 2005; Völlm et al. 2006). Several functional magnetic resonance imaging (fMRI) studies have shown deviant activation in the STS/middle temporal gyrus region in ASD individuals and their first-degree relatives during mental state attribution (e.g., Castelli et al. 2002; Baron-Cohen et al. 2006). Structural alterations in the STS and middle temporal gyrus may be related to these deviant activation patterns and associated social-cognitive deficits in ASD.

Our finding of reduced GM volume in the ACC in the ASD group replicates results from earlier studies (Hadjikhani et al. 2006; Haznedar et al. 2000). The ACC has been implicated in various higher-order social-emotional and cognitive functions (Allman et al. 2001), such as response monitoring and affective regulation. Several fMRI studies on executive function or socio-emotional processing have reported altered ACC activation along with poorer task performance in ASD subjects (Di Martino et al. 2009). It should be kept in mind, however, that functional and structural alterations in the ACC are not restricted to ASD, but have also been observed in other psychiatric conditions, e.g., in schizophrenia (Pauly et al. 2008). It seems plausible that alterations in the ACC might be associated with (psychiatric) symptoms, like executive or socio-emotional deficits, rather than with specific diagnoses.

### Alterations in age-related changes in regional grey matter volumes

Investigation of age-related changes of GM volumes in this cross-sectional study revealed complex region- and hemisphere-specific alterations in ASD, indicating disturbances in developmental trajectories. Most of the regions where alterations were found belong to an anatomically and functionally closely connected network that underpins social-cognitive and emotional functions (Müller et al. 2012; Swenson 2006). With this respect, it is of interest that structural connectivity within this network is disrupted in ASD, e.g., as evidenced in a disruption of WM pathways linking temporal lobe structures including the amygdala (Ameis et al. 2011).

Our results help to reconcile some of the contradictory findings on structural changes in ASD, since previous studies may have yielded differential results depending on the age group investigated. In most cortical regions where differences were found, GM volume curves in ASD subjects were shifted leftwards along the age axis relative to controls. Thus, taking a cross-sectional view, peak GM volume in these areas was observed earlier in ASD relative to control subjects.

This finding is consistent with previous reports of early brain overgrowth in young ASD subjects (Amaral et al. 2008, but see Raznahan et al. 2010). Interestingly, the leftward shift of GM volume curves in ASD is in stark contrast to brain maturation curves observed in ADHD, which are characterized by a rightward shift along the age axis, indicating a delay in brain development (Shaw et al. 2007).

Changes in amygdala development are one of the most striking findings, in particular as the data suggest differential developmental patterns for the two hemispheres. Our finding can be brought in line with a recent cross-sectional study in healthy individuals, which found a non-linear increase in amygdala volume between the ages of 8 and 30 (Ostby et al. 2009). Regional GM volume curves for both the right and left amygdala in the ASD group were shifted to the left compared to controls, indicating earlier (over-)growth in autistic individuals. Notably, in contrast to the pattern observed for the left amygdala, GM volume curves for the right amygdala converged with increasing age.

Our results corroborate and extend data by Schumann et al. (2004), who reported differential alterations of amygdala volume in ASD subjects relative to controls depending on the age group investigated. Age effects on amygdala alterations in ASD have recently also been demonstrated in a meta-analysis reporting amygdala enlargements in younger, but not older, ASD subjects (Stanfield et al. 2008). Amongst other regions, the amygdala is interconnected with many brain regions that are involved in emotional and social processing, such as the insula, the orbital and medial prefrontal cortex (Gray 1999). Some authors suggest that early abnormalities in the amygdala may account for social impairments associated with ASD (Schultz 2005). This claim is supported by a longitudinal MRI study that reported an association between amygdala enlargement at age 3–4 years and worse social functioning at age 6 years (Munson et al. 2006). Future longitudinal studies spanning childhood and adulthood may help to further elucidate the relationship between amygdala maldevelopment in ASD and social deficits over the developmental course.

Hemispheric-specific alterations in age-related amygdala development in ASD subjects, as found in the present study, might be associated with functional differences between the right and left amygdala. A meta-analysis of functional neuroimaging studies found that amygdala activation is lateralized to the left for verbally presented emotional stimuli (Costafreda et al. 2008). The extraction of emotion based on language-related material is particularly difficult for individuals with ASD and remains difficult for affected adults (Rutherford et al. 2002). With this respect, persisting difficulties in understanding language-mediated emotions might be related to alterations in GM development of the left amygdala across the life-span. By contrast, processing emotional information in language-independent forms (e.g., when conveyed via the face) is often trained extensively in intervention programs (Herbrecht et al. 2009), what might perhaps result in a normalisation of right amygdala development with increasing age.

Furthermore, the results from our study indicate earlier GM maturation in ASD in the left septal nucleus and right TPJ. The left septal nucleus has been implicated in the pathophysiology of ASD (Bauman and Kemper 1985). Importantly, this region is a part of the limbic circuitry and has reciprocal connections to the amygdala and the hippocampus (Leutgeb and Mizumori 1999; Swenson 2006). To our knowledge, our study is the first to report age-related changes in septal nucleus development.

The TPJ is a multimodal association area, which integrates information from the thalamus and from auditory, visual, sensory and limbic areas. It is anatomically closely connected to the temporal lobes and prefrontal regions (Decety and Lamm 2007). Results from a comprehensive study in healthy children and young adolescents suggest that the TPJ matures late, as evidenced by a relatively late loss of GM in this brain structure compared to brain regions implicated in more basic functions (Gogtay et al. 2004). Functional imaging studies suggest that the TPJ is relevant not only for various social-cognitive processes but also for lower-level attentional mechanisms which might be a crucial prerequisite for higher-level social cognition (Decety and Lamm 2007). fMRI studies in ASD individuals involving empathic responding (Schulte-Rüther et al. 2011) and mental state attribution (Castelli et al. 2002) reported deviant activation in the TPJ in ASD individuals compared to controls. Our findings extend the observed pathology to the structural domain and may provide another anatomical correlate for the persisting social-cognitive deficits in ASD.

Finally, changes in GM development were observed in the middle cingulate cortex and right precentral gyrus. The GM volume curve in the middle cingulate cortex was again shifted to the left in ASD subjects and diverged further away from typical development with increasing age. In line with our study, a study by Shaw et al. (2008) reported a negative quadratic GM-growth trajectory in the supracallosal portion of the cingulate in 375 children and adults. Amongst other regions, the middle cingulate cortex is anatomically connected to the amygdala, the insula and the orbitofrontal cortex, as well as to the motor and premotor cortex (Morecraft and Van Hoesen, 1992, 1998, 2007). In healthy individuals, this region is preferentially recruited in response to self-related information (Lombardo et al. 2010). fMRI studies have found reduced middle cingulate cortex activation in ASD subjects during self-related processing, which might be linked to social impairments in the disorder (Chiu et al. 2008; Lombardo et al. 2010). In future studies, it would be of great interest to investigate the role of atypical structural development of the middle cingulate cortex in ASD with regard to deviant self-representation and social deficits.

Group differences in GM development of the precentral gyrus were particularly striking, since in this part of the motor cortex, linear volume decreases with age were observed in ASD subjects while GM change in controls followed a U-shaped pattern. Our finding in controls is in line with a study that reported a U-shaped age-related pattern in GM density in 176 subjects across the life span (Sowell et al. 2003). The precentral gyrus is anatomically connected to the postcentral area and prefrontal regions, and also forms connections with parietal and temporal areas (Schubotz et al. 2010). It should be pointed out that volume changes as revealed by our VBM analysis have to be interpreted in terms of relative changes in local GM volume with respect to overall brain size. We thus tentatively suggest that the observed volume increase in the precentral gyrus in controls during early and middle adulthood may reflect structural plasticity of the motor cortex related to motor skill learning (Gaser and Schlaug 2003), presumably interacting with maturational processes. Conversely, motor impairments in ASD, such as deficits in fine and gross motor skills and motor planning, are well documented (Hughes 1996; Jansiewicz et al. 2006). The maldevelopment of motor cortices demonstrated by our data may thus relate to or provide a structural underpinning of the motor impairments in ASD.

It is of interest that some of the brain regions where age-related changes in the ASD group were found to show histological abnormalities in post-mortem ASD brains (Bauman and Kemper 1985; Casanova et al. 2006; Schumann and Amaral 2006). Casanova et al., e.g., reported abnormalities in minicolumnar morphology in post-mortem brains from ASD individuals. To date, both the causes for such histological abnormalities as well as for changes in age-related structural development in ASD remain unclear. Recently, two alternative explanations for changes in brain maturation patterns in ASD have been put forward (Raznahan et al. 2010). One explanation posits that genetically mediated abnormalities (e.g., in synaptic function) and environmental risk factors associated with ASD directly impact on structural maturation patterns in affected individuals. Another possibility is that some alterations in structural maturation are secondary to the disorder, in that age-related changes in GM growth are the result of an “underuse” of certain brain regions with an initial normal developmental pathway.

Our finding of non-linear age-related GM changes in controls and ASD subjects in most regions where group differences were found is in line with cross-sectional and longitudinal studies in healthy individuals which report curvilinear relationships between GM volume and age for most cortical regions across the life span (Giedd et al. 1999; Sowell et al. 2003).

The maturational trajectory of GM development is highly region-specific and closely parallels changes in behaviour and functions associated with certain brain structures (Gogtay et al. 2004). Our finding of continued GM development in the control group beyond adolescence in regions that predominantly support social-cognitive abilities can be brought in line with studies showing age-related changes in these abilities and the underlying functional network into adulthood (Choudhury et al. 2006; Greimel et al. 2010). In addition, in line with our study, social-cognitive skills in ASD subjects improve from childhood to adulthood (Schwenck et al. 2012; Seltzer et al. 2004), although affected adults do not seem to “catch up” in most domains (but see, e.g., Dziobek et al. 2008).

It should be noted that, unlike other studies, we did neither find enlarged caudate nucleus volumes (Haznedar et al. 2006; Hollander et al. 2005; Langen et al. 2009; Rojas et al. 2006) nor divergent GM development of the caudate nucleus (Langen et al. 2009; McAlonan et al. 2002) in ASD subjects. However, many of these studies used a ROI-based approach, which is certainly more sensitive to findings in an a priori specified region than the whole-brain VBM approach taken in the present investigation. In particular if there are distributed or small changes in a structure such as the caudate, there may be no single voxel in a General Linear Model analysis that survives statistical thresholding even though the total volume may be decreased. Moreover, the divergent finding of the present investigation may also be explained by differences between the studies in the clinical characteristics and the age group investigated.

Moreover, it should be discussed that we did not find significant correlations between regional GM volumes and the ADOS-G. This finding might be explained by the fact that the ADOS-G is not particularly appropriate for deriving a quantitative measure of impairment. In future studies, it would be worthwhile to apply measures which are better suited for quantitative assessment of autistic psychopathology (e.g., the Social Responsiveness Scale; Constantino 2002) for investigation of brain-behaviour relationships.

The present study globally assessed whether age-related regional GM volume curves differed between ASD individuals and controls with regard to linear, quadratic or compound effects of age. Based on the findings of the present investigation, it would be valuable in future studies to state and test more specific hypotheses regarding changes in GM development in ASD, e.g., regarding the age at which the most severe changes in GM development occurs.

Some putative limitations of our study need to be considered. Most importantly, a longitudinal design (Schumann et al. 2010) might seem more appropriate to investigate developmental trajectories of GM than the cross-sectional approach taken (Kraemer et al. 2000). This, however, is difficult to achieve in a cohort of ASD subjects as large as our sample. Moreover, only ASD subjects with an IQ >70 were studied to allow for homogenous groups. By necessity, the generalizability of our findings to the whole spectrum of autistic disorders may be limited, particularly as there is evidence that ASD subtypes may be associated with distinct neuroanatomical abnormalities (Nordahl et al. 2007; Scott et al. 2009). In the present study, a large number of subjects were included. However, in light of the broad age range studied, it would have been favourable to include even more subjects, especially in the upper age range. This would also allow to investigate different age groups separately, although, particularly in adulthood, biologically meaningful age brackets are still difficult to define.

## Conclusion

The present study provides important new insights into the structural pathophysiology of ASD. Our findings indicate that ASD is characterised by complex changes in GM developmental trajectories of several brain regions. These brain regions belong to neural networks that underpin social-cognitive and motor functions previously demonstrated to be impaired in ASD. Changes in GM development present during childhood and persisting into adolescence and adulthood are likely to contribute to social-cognitive and motor impairments in individuals affected by ASD.

In future longitudinal studies spanning childhood and adulthood, it would be of particular interest to relate clinical outcome in ASD to individual differences in brain structure and brain development. In the long run, such an approach might prove important to individualise and optimise treatment strategies in ASD individuals.

## Electronic supplementary material

Below is the link to the electronic supplementary material.

Appendix 1 (DOC 35 kb)

Appendix 2 (DOC 57 kb)
