# Supplementary material for: Changes in grey matter development in autism spectrum disorder
Source: Brain Struct Funct. 2012 Jul 10;218(4):929–42. doi: 10.1007/s00429-012-0439-9 (PMC3695319; doi:10.1007/s00429-012-0439-9)
Supplement: Supplementary file 1 — Appendix 1 (DOC 35 kb) [file 429_2012_439_MOESM1_ESM.doc]

**Appendix 1**

**Changes in grey matter development in autism spectrum disorder**

**Journal name: Brain Structure and Function**

Ellen Greimel, Barbara Nehrkorn, Martin Schulte-Rüther, Gereon R. Fink, Thomas Nickl-Jockschat, Beate Herpertz-Dahlmann, Kerstin Konrad & Simon B. Eickhoff

**Corresponding author**:

Ellen Greimel, Department of Child and Adolescent Psychiatry, Psychosomatics and Psychotherapy, University Hospital Munich, Germany

E-mail: [Ellen.Greimel@med.uni-muenchen.de](mailto:Ellen.Greimel@med.uni-muenchen.de), Ph.: +49 89 51605914, Fax: +49 89 51605902

**Appendix 1:** Probabilistic empirical Bayes algorithm

The algorithm calculates the conditional distribution for the parameter estimates (across subjects) at each voxel using the variance across voxels as Bayesian prior (Friston et al. 2002a; Friston et al. 2002b; Friston and Penny 2003a; Friston and Penny 2003b; Friston et al. 2008)(Friston et al. 2008; Friston et al. 2002a; Friston and Penny 2003a; Friston et al. 2002b; Friston and Penny 2003b). The resulting posterior probability maps were thresholded at a probability of 0.99 for an effect size greater than the prior standard deviation (γ threshold). Thus, only those voxels were considered as being different between groups, whose parameter estimates were larger than γ with at least 99% confidence. The rationale for using the prior standard deviation as the effect size threshold γ is that it equates to a ‘‘background noise level’’ that is generic to the brain as a whole. The chosen threshold allows directing Bayesian inference to only show those voxels that are almost certainly different between groups, thereby accounting for the background noise level.

References

Friston K, Chu C, Mourao-Miranda J, Hulme O, Rees G, Penny W, et al. (2008) Bayesian decoding of brain images. Neuroimage 39:181-205

Friston KJ, Glaser DE, Henson RNA, Kiebel S, Phillips C, Ashburner J (2002a) Classical and Bayesian Inference in Neuroimaging: Applications. Neuroimage 16(2):484-512

Friston KJ, Penny W (2003a) Posterior probability maps and SPMs. Neuroimage 19:1240-9

Friston KJ, Penny W, Phillips C, Kiebel S, Hinton G, Ashburner J (2002b) Classical and Bayesian Inference in Neuroimaging: Theory. Neuroimage 16(2):465-83

Friston KJ, Penny WD (2003b) Classical and Bayesian inference. In: Frackowiak RS, Friston KJ, Frith CD, Dolan RJ, Price CJ, Ashburner J, Penny WD, Zeki S, (eds) Human Brain Function. Academic Press
