# Supplementary material for: Changes in grey matter development in autism spectrum disorder
Source: Brain Struct Funct. 2012 Jul 10;218(4):929–42. doi: 10.1007/s00429-012-0439-9 (PMC3695319; doi:10.1007/s00429-012-0439-9)
Supplement: Supplementary file 2 — Appendix 2 (DOC 57 kb) [file 429_2012_439_MOESM2_ESM.doc]

**Appendix 2**

**Changes in grey matter development in autism spectrum disorder**

**Journal name: Brain Structure and Function**

Ellen Greimel, Barbara Nehrkorn, Martin Schulte-Rüther, Gereon R. Fink, Thomas Nickl-Jockschat, Beate Herpertz-Dahlmann, Kerstin Konrad & Simon B. Eickhoff

**Corresponding author**:

Ellen Greimel, Department of Child and Adolescent Psychiatry, Psychosomatics and Psychotherapy, University Hospital Munich, Germany

E-mail: [Ellen.Greimel@med.uni-muenchen.de](mailto:Ellen.Greimel@med.uni-muenchen.de), Ph.: +49 89 51605914, Fax: +49 89 51605902

**Appendix 2**

*Equations and their parameters for the second-order polynomial fit of the age-related changes in local grey matter (GM) volume at the respective peak voxels*

Left amygdala:

ASD-GM volume = -0.0181*Age^2 + 1.0242*Age + 25.6790
HC-GM volume = -0.0142*Age^2 + 1.0390*Age + 21.2201

Right temporoparietal junction:

ASD-GM volume = -0.0192*Age^2 + 1.0187*Age + 26.2649
HC-GM volume = -0.0147*Age^2 + 0.9441*Age + 24.1790

Left septal nuclus:

ASD-GM volume = -0.0136*Age^2 + 0.7934*Age + 20.0694#
HC-GM volume = -0.0088*Age^2 + 0.5927*Age + 20.5356

Middle cingulate cortex:

ASD-GM volume = -0.0168*Age^2 + 0.9650*Age + 26.6037
HC-GM volume = -0.0086*Age^2 + 0.7571*Age + 25.8176

Right amygdala:

ASD-GM volume = -0.0075*Age^2 + 0.6318*Age + 35.2577
HC-GM volume = -0.0112*Age^2 + 0.9272*Age + 27.9564

Right precentral gyrus:

ASD-GM volume = -0.0004*Age^2 + -0.1390*Age + 28.6455
HC-GM volume = 0.0183*Age^2 + -1.2672*Age + 42.9611

ASD=autism spectrum disorder; HC=healthy controls.

**Supplementary analyses**

*Reanalysis of the data restricted to subjects scanned on the 1.5 Tesla system*

In the reanalysis, the only difference that emerged with respect to regional GM volume across age was that GM volume reduction in the STS in ASD subjects was restricted to the right hemisphere and thus was not found bilaterally as in the analysis including all N=98 subjects.

Moreover, a slight difference emerged with respect to group differences in age-effects in the way that GM volume curves in the right amygdala did not converge with increasing age as described in our analysis including all N=98 subjects. Importantly, however, the result that the GM volume curve in this region exhibited a leftward shift along the age axis in ASD subjects remained the same.

*Analysis of medication effects*

To examine potential effects of medication, regional GM volumes were compared between ASD subjects that were medicated at time of testing and unmedicated ASD subjects. Modulated GM images of unmedicated and medicated ASD subjects were entered in a second-level Bayesian mixed effects model with group as condition (medicated vs. unmedicated). To control for potential confounding variables, TBV, age (linear and quadratic age regressor: age and age in years squared, respectively), full-scale, verbal and performance IQ were entered as covariates into the same model. This analysis did not reveal any significant group differences in regional GM volumes when thresholded at a posterior probability of P>.99. Importantly, also when the threshold was lowered to P>0.5 (i.e., 50% chance of true effects), no significant differences emerged.

Moreover, examination of group differences in linear, quadratic or compound effects of age on regional GM volumes based on an ANCOVA with two regressors specifying linear and quadratic effects of age as well as regressors reflecting TBV, full-scale, verbal and performance IQ did not reveal any significant effects of medication at the pre-specified threshold of p<.05 (FDR-corrected). When the threshold was lowered to an exploratory p<0.001 uncorrected, again no significant differences emerged between medicated and unmedicated ASD subjects.

*Analysis of the effect of diagnostic group*

To examine potential differences between diagnostic groups, regional GM volumes were compared between subjects with Asperger syndrome and with high-functioning autism. For this aim, modulated GM images of these subjects were entered in a second-level Bayesian mixed effects model. To control for potential confounding variables, TBV, age (linear and quadratic age regressor: age in years and age in years squared, respectively), full-scale, verbal and performance IQ were entered as covariates into the same model. This analysis did not reveal any significant group differences in regional grey matter volumes, even when thresholded at a posterior probability of .50.

Moreover, to examine potential differences between diagnostic groups on age-related changes of GM volume, a second-level random effects group analysis was conducted based on an ANCOVA. Two regressors specifying linear and quadratic effects of age as well as regressors reflecting TBV, full-scale, verbal and performance IQ were included as covariates into the model. F-contrasts did not reveal significant differences between the groups in age-related changes of GM volume, even when applying a liberal statistical threshold (p=.001 uncorrected).

*Estimates of effect sizes (Cohen´s d) for group differences in regional grey matter volumes*

*
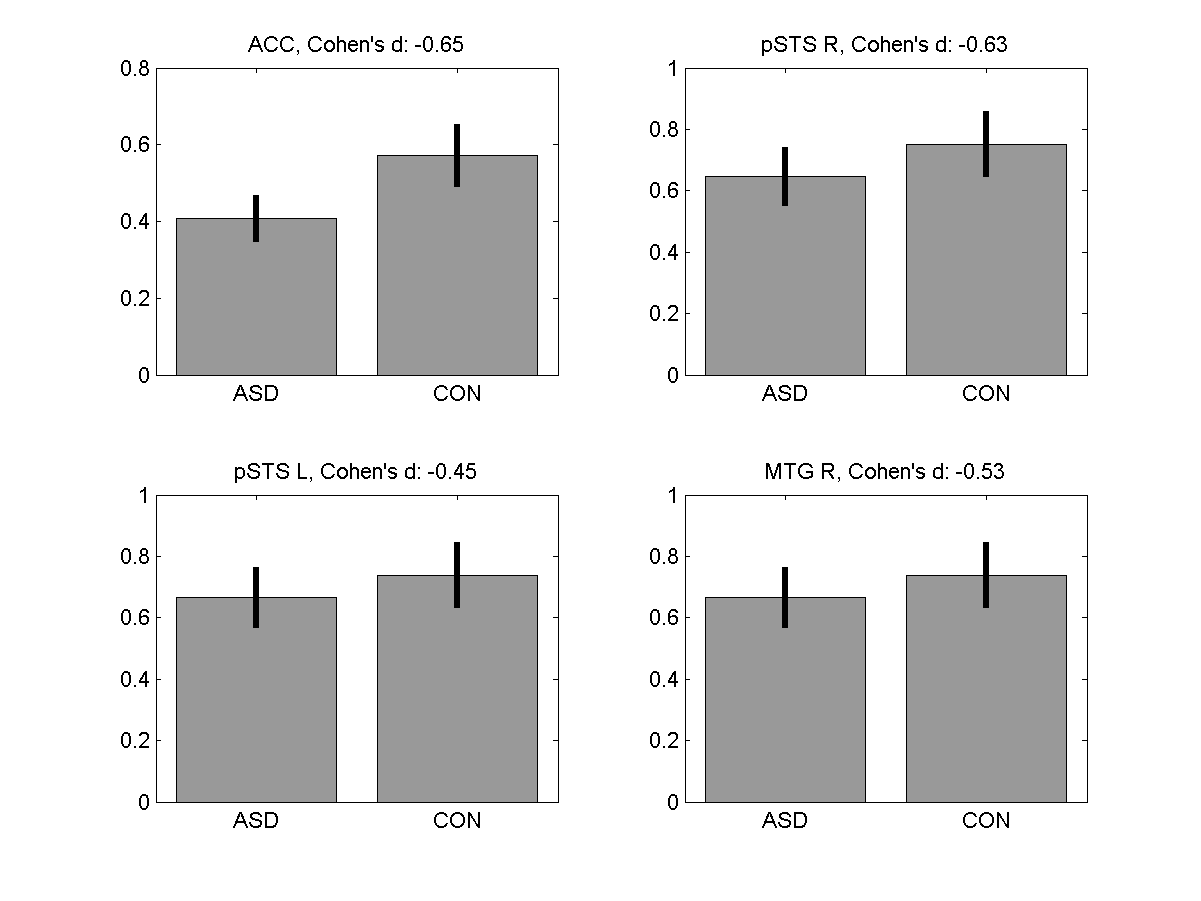
*

Abbreviations: ACC=anterior cingulate cortex; pSTS R/L=right/left posterior superior temporal sulcus (local maxima in the right and left middle temporal gyrus); MTG R=right middle temporal gyrus; ASD=autism spectrum disorder group; con=control group.
